# Mesoporous Cu–Cu_2_O@TiO_2_ heterojunction photocatalysts derived from metal–organic frameworks†

**DOI:** 10.1039/d0ra01327g

**Published:** 2020-04-09

**Authors:** Wenling Zhao, Chengcheng Liu

**Affiliations:** Institute of Molecular Sciences and Engineering, Institute of Frontier and Interdisciplinary Science, Shandong University Qingdao 266237 P. R. China chengcheng.liu@sdu.edu.cn

## Abstract

This study reveals a unique Cu–Cu_2_O@TiO_2_ heterojunction photocatalyst obtained with metal–organic framework as the precursor, which can be utilized in dye photodegradation under visible light irradiation. The composition, structure, morphology, porosity, optical properties and photocatalytic performance of the obtained catalysts were all investigated in detail. The Cu–Cu_2_O@TiO_2_ nanocomposite is composed of lamellar Cu–Cu_2_O microspheres embedded by numerous TiO_2_ nanoparticles. Methylene blue, methyl orange and 4-nitrophenol were used as model pollutants to evaluate the photocatalytic activity of the Cu–Cu_2_O@TiO_2_ nanocomposite for dye degradation under visible light irradiation. Nearly 95% decolourisation efficiency of Methylene blue was achieved by the Cu–Cu_2_O@TiO_2_ photocatalyst within 3 h, which is much higher than that of TiO_2_ or Cu_2_O catalysts. The excellent photocatalytic activity was primarily attributed to the unique MOF-based mesoporous structure, the enlarged photo-adsorption range and the efficient separation of the charge carriers in the Cu–Cu_2_O@TiO_2_ heterojunction.

## Abstract 2

Cu–Cu_2_O@TiO_2_ heterojunction photocatalyst derived from a metal–organic framework shows high photocatalytic activity for dye degradation under visible light irradiation.

## Graphical abstract

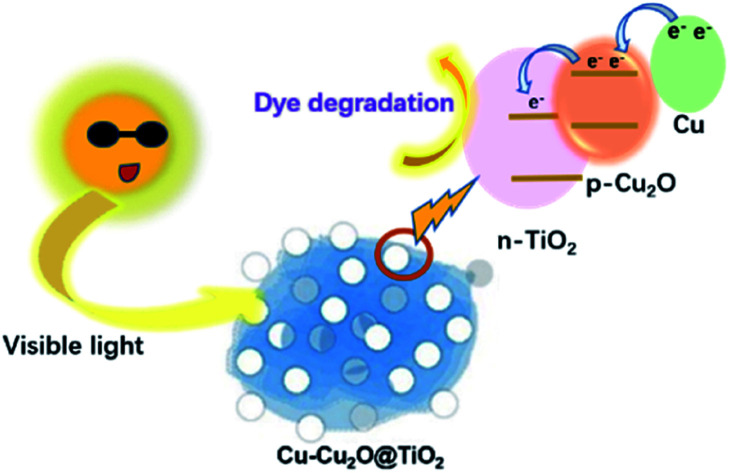

## 1. Introduction

Photocatalysis has emerged as an economical and environmentally benign reaction route and has shown potential in tackling resource depletion in recent years.^1,2^ In this process, the light energy can be converted into the energy required for chemical reactions. Semiconductors, *e.g.*, TiO_2_, Cu_2_O, Cu_2−*x*_Se, and g-C_3_N_4_*etc.*, with relatively low cost, high catalytic activity and stability are commonly used as photocatalysts.^3–6^ Silver based nanocomposites are also photocatalysts for dye degradation.^7,8^ Titanium dioxide (TiO_2_), known as one of the most important n-type semiconductors, has been recognised as a photocatalyst owing to its own characteristics such as good photostability, low cost and environmental friendliness.^3,4^ Due to its wide bandgap (anatase, 3.2 eV), TiO_2_ can only be excited by ultraviolet light for photocatalytic reactions.^9^ However, ultraviolet light only accounts for 5% of the solar spectrum, while visible light accounts for 48% of the total light energy.^10^ At the same time, the photogenerated positive and negative charge carriers, electron–hole pairs, tend to recombine in single-phase semiconductors, resulting in the low efficiency of photocatalytic reactions. To overcome these shortcomings, a great number of efforts have been devoted to modifying the TiO_2_ nanostructure surface or combining single-phase TiO_2_ with non-metals, metals, and organic molecules to form semiconductor composites. It has been reported that the combination of two or more desirable semiconductors or metal nanoparticles is an effective method to create nanocomposites with higher photocatalytic activity.^11,12^ Because the photogenerated electrons are found to be transferable between them in a composite, which can significantly promote the separation of electron–hole pairs and finally improve photocatalytic efficiency. It is well known that narrow-bandgap semiconductor can serve as the primary absorber of visible light in a coupled semiconductor. Among narrow-bandgap semiconductors, Cu_2_O is a naturally occurring p-type semiconductor with a wide range of applications such as photocatalytic water splitting, gas sensing, and degradation of organic pollutants.^13–15^ Different from TiO_2_, Cu_2_O can be easily excited by visible light to generate electron and hole pairs on account of its lower bandgap energy (2.17 eV).^16^ The matched band structures of Cu_2_O and TiO_2_ are sufficient to promote the physical separation of charge carriers, and also significantly inhibit the recombination rate in their nanocomposite, thereby increasing the light-harvesting efficiency and enhancing photocatalytic activity.^17^ Moreover, the use of Cu coupled to Cu_2_O and TiO_2_ has shown better performance owing to the surface plasmon resonance (SPR) effect of Cu nanoparticles.^18,19^ However, according to the literature, the synthesis of Cu–Cu_2_O–TiO_2_ nanocomposites requires sophisticated equipment, and it is very difficult to obtain a well-designed unique three-dimensional morphology of the nanocomposites. For this reason, the development of a controllable and straightforward synthetic route for Cu–Cu_2_O–TiO_2_ photocatalysts with novel structures and remarkable photocatalytic performance under visible light remains a challenge.

Catalysts with controlled porosity and morphology have shown great advantages in catalysis owing to their good mass transfer property, large surface area and abundant reactive sites.^20^ Due to its unique porous structure and tuneable organic linkers/metal clusters, MOFs have attracted considerable attention as precursors to derive various useful porous catalytic materials by thermal or chemical treatments. For example, thermal treatment of MOFs in an inert atmosphere can produce highly porous carbon with a high specific surface area (∼3000 m^2^ g^−1^).^21^ Besides, direct heat treatment of MOF in the optimised heating conditions in a nitrogen or air atmosphere can lead them to decompose into their corresponding metal oxides.^22^ In general, the derivative metal oxides have the same structure as the parent MOFs, and show much higher surface area than metal oxides produced by other methods. The high surface area, controllable porous architectures and tuneable composition of MOF-derived metal oxides along with the possibilities to combine them with different catalytic active metal oxides make them attractive candidates for the catalytic applications.^23^ Over the years, metal-oxide semiconductors (MOS) have received the extensive concern on account of their high photo-response activity and nontoxicity in the application of solar energy conversion and environmental pollution reduction.^24^

Based on the above considerations, this work utilized cheap copper MOFs, NOTT-100 (Cu), as precursor to develop a kind of photocatalytic-active porous Cu–Cu_2_O@TiO_2_ triple junction nanocomposite, which was built by Cu–Cu_2_O microspheres coated by a great many TiO_2_ nanoparticles. To investigate the structure and morphology of Cu–Cu_2_O@TiO_2_ nanocomposite and its optical properties, XRD, SEM, UV-vis, *etc.* were conducted. The Cu–Cu_2_O@TiO_2_ shows good photocatalytic activity in organic pollutants degradation under visible-light illumination. The synergic effect endows the composite higher performance and the enhancement of the catalysis was attributed to the special heterojunction structure and the mesoporous structure.

## 2. Results and discussion

### 2.1 Structure and morphology

**Scheme 1:**
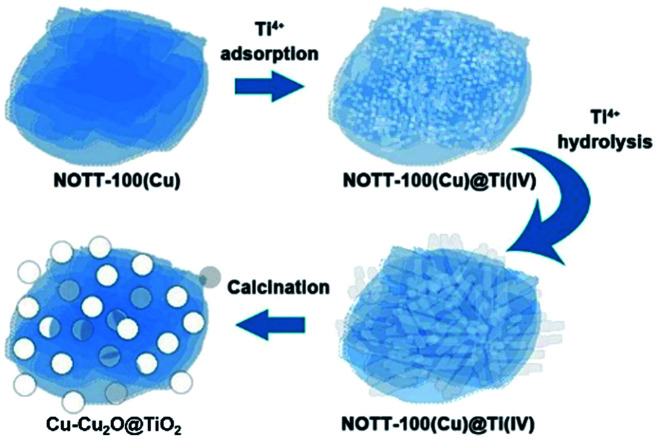
Synthesis procedure of Cu–Cu_2_O@TiO_2_ nanocomposite.

In this project, Cu–Cu_2_O@TiO_2_ nanocomposite was synthesised by an easy two-step method. NOTT-100(Cu) lamellar microstructures were obtained firstly using the solvothermal method, and then tetra-*n*-butyl orthotitanate, the Ti(iv) precursors, were adsorbed on the porous surface of MOF microstructures, taking advantage of porosity of MOFs and strong hydrolysis ability of Ti(iv) precursors to produce the Cu–Cu_2_O@TiO_2_ nanocomposite after calcination (Scheme 1). Firstly, tetra-*n*-butyl orthotitanate was dissolved in the NOTT-100(Cu) suspension in *n*-hexane. Abundant Ti^4+^ cations would be diffused and adsorbed onto the surfaces of the porous NOTT-100(Cu) and partially hydrolysed with water molecules on the MOFs. After the separation of solvent *n*-hexane, Ti^4+^ cations further hydrolysed quickly with the moisture in the air. With the calcination, NOTT-100(Cu) totally decomposed to Cu_2_O at 550 °C in 2 h. Then part of the Cu_2_O decomposed to Cu when heated continuously to 4 h (Fig. S2†). Hence Cu_2_O and Cu coexist under the adopted calcination temperature and time conditions. Besides, a large number of TiO_2_ nanoparticles were formed from hydrolysed products, obtaining TiO_2_ nanoparticles grown on Cu–Cu_2_O microspheres.

This adsorption and hydrolysis procedure did not influence the unique structure of NOTT-100(Cu) as shown in Fig. S1.† The calcination temperature of 550 °C and duration of 4 hours are selected as the optimised calcination condition for Cu–Cu_2_O formation without CuO (Fig. S2†). For this sample, all diffraction peaks in the XRD pattern are almost consistent with each value of the standard patterns of anatase phase TiO_2_ (JCPDS card no. 21-1272), Cu (JCPDS card no. 04-0836) and Cu_2_O (JCPDS card no. 05-0667), indicating the good-crystalline character of the samples.

**Fig. 1:**
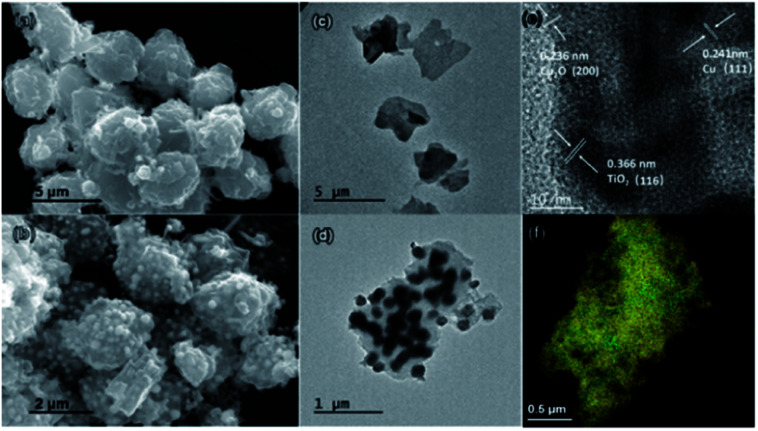
SEM and TEM images of the NOTT-100(Cu) (a) and (c); Cu–Cu_2_O@TiO_2_ (b) and (d). HRTEM images of the Cu–Cu_2_O@TiO_2_ (e). EDS mapping of O (red), Ti (green) and Cu (yellow) in Cu–Cu_2_O@TiO_2_ (f).

In Fig. 1, SEM and TEM images illustrate the morphologies and microstructures of NOTT-100(Cu) and Cu–Cu_2_O@TiO_2_, respectively. It can be clearly seen that the Cu–Cu_2_O@TiO_2_ nanocomposite consists of flower-like lamellar microspheres with a diameter of about 2.5 μm keeping the primary structure of NOTT-100(Cu), and a large number of nanoparticles covered on the surface of lamellar microstructures. In addition, Fig. 1e shows the HRTEM image of the Cu–Cu_2_O@TiO_2_, from which it can be seen that the lattice space of 0.366 nm corresponds to (116) planes of TiO_2_. The lattice space of 0.241 nm corresponds to (111) planes of Cu, and the lattice space of 0.236 nm corresponds to (200) planes of Cu_2_O. EDS mapping shows the element distribution of O, Ti and Cu in Cu–Cu_2_O@TiO_2_ (Fig. 1f and S3†). These characterizations demonstrate that almost all TiO_2_ nanoparticles attached to the Cu–Cu_2_O surface are separately and evenly distributed. Hence, it can be concluded that the as-prepared rough Cu–Cu_2_O@TiO_2_ nanocomposite is composed of the MOF-like lamellar Cu–Cu_2_O microspheres embedded by a great many TiO_2_ nanoparticles.

The porosity of the Cu–Cu_2_O@TiO_2_ sample was investigated by N_2_ adsorption–desorption isotherm patterns (Fig. S4†). The isotherm is similar to type-IV IUPAC isotherm which indicates the Cu–Cu_2_O@TiO_2_ sample is a kind of mesoporous material. The average pore diameter is determined about 7 nm. It is reported that mesoporous structure has a large specific surface area, providing a large number of active centers for the reaction. Moreover, the existence of mesoporous structure is conducive to multiple light scattering or reflection, thus enhancing the capture of exciting light and improving the photocatalytic activity.^25–27^ The BET surface area and the pore volume of Cu–Cu_2_O@TiO_2_ are 187 m^2^ g^−1^ and 0.298 cm^3^ g^−1^ respectively. The BET surface is much higher than the reported Cu–Cu_2_O–TiO_2_ composite (50m^2^ g^−1^) owing to the porous properties.^28^ The mesopores are assumed to result from the loss of organic ligands in MOF during calcination. Therefore, the Cu–Cu_2_O@TiO_2_ nanocomposite is revealed to have an excellent porous property, which can be another advantage for the catalytic application.

### 2.2 XPS analysis

**Fig. 2:**
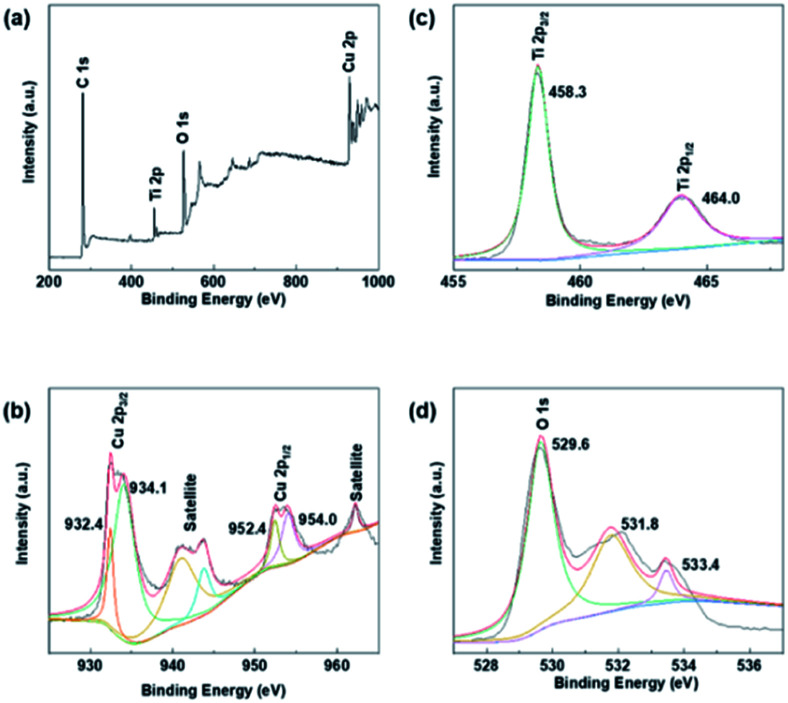
XPS spectra of Cu–Cu_2_O@TiO_2_: (a) XPS survey spectrum, (b) Cu 2p spectrum, (c) Ti 2p spectrum and (d) O 1s spectrum.

XPS studies were conducted over the Cu–Cu_2_O@TiO_2_ in order to understand the chemical state and chemical environment of the Cu and Ti elements of the composite. Fig. 2a shows the XPS survey spectrum for Cu–Cu_2_O@TiO_2_ composites, which demonstrates the existence of Cu, Ti, O and C elements in the sample. The spectra were calibrated with C 1s as standard. Fig. 2b shows the high resolution XPS spectrum in the region of Cu 2p for Cu–Cu_2_O@TiO_2_. The main peak centered at 932.4 and 952.4 eV of Cu (2p_3/2_) and Cu (2p_1/2_) are readily assigned to either Cu(0) or Cu(i). The two binding energy peaks located at 934.1 and 954.0 eV with two extra shake-up satellites were assigned to Cu (2p_3/2_) and Cu (2p_1/2_) of Cu(ii).^29^ This was commonly attributed to the oxidation of Cu(i) during sample preparation for analysis as there is no CuO shown in the XRD spectrum.^30^ The high resolution XPS spectrum of Ti 2p in Cu–Cu_2_O@TiO_2_ (Fig. 2c) centered in 458.3 and 464.0 eV can be assigned to Ti (2p_3/2_) and Ti (2p_1/2_) for Ti(iv).^31^

### 2.3 Optical properties and photocatalytic performance

**Fig. 3:**
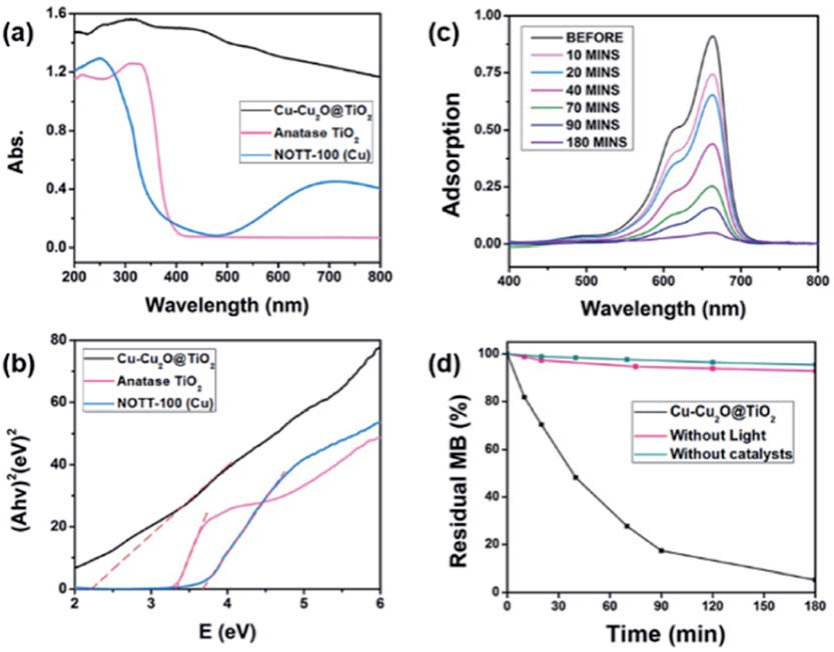
(a) UV-vis diffuse-reflectance spectra and (b) Tauc plots of (*Ahν*)^2^*versus* (*hν*) of Cu–Cu_2_O@TiO_2_, TiO_2_(anatase) and NOTT-100(Cu), respectively; (c) UV-vis absorption spectra for MB solution in the presence of Cu–Cu_2_O@TiO_2_ nanocomposite under the visible light irradiation at different time intervals; (d) MB residual under different conditions.

The UV-vis diffuse reflectance spectroscopy was employed to investigate the optical properties of the Cu–Cu_2_O@TiO_2_ sample. The UV-vis absorption spectra of pure Cu–Cu_2_O@TiO_2_ nanocomposite, pure commercially available TiO_2_ (anatase) nanoparticles, pure NOTT-100(Cu) are shown in Fig. 3a. It can be clearly seen that the pure TiO_2_ presents strong absorption of ultraviolet light at wavelengths below 400 nm. As desired, the Cu–Cu_2_O@TiO_2_ nanocomposite exhibits much stronger absorption, especially in wavelengths ranging from 400 to 600 nm, which could be attributed to the interplay of Cu_2_O and TiO_2_ and the narrow band-gap of Cu_2_O.

Using the Tauc plot of (*Ahν*)^2^*versus* photon energy (*hν*), the optical bandgap of the direct allowed semiconductor could be roughly determined.^32^ As shown in Fig. 3b, the measured bandgap of pure TiO_2_ and pure NOTT-100(Cu) is about 3.34 eV and 3.67 eV, respectively. As the coupling of Cu–Cu_2_O has a significant effect on the band-gap energy of TiO_2_, the estimated band-gap of the Cu–Cu_2_O@TiO_2_ nanocomposite is 2.25 eV, which is much lower than band-gap of either pure TiO_2_ or pure NOTT-100(Cu) but little higher than that of reported pure Cu_2_O (2.17 eV).^33^ These results indicate that the interfaces of Cu–Cu_2_O and TiO_2_ are combined intimately, and the band edges achieve good matching between the two semiconductors. Hence, the visible light harvest and high-efficient separation of electron and hole pairs are more likely to accomplish in the Cu–Cu_2_O@TiO_2_ nanocomposite.

In this work, methylene blue (MB), methyl orange (MO) and 4-nitrophenol (4-NP) were utilised as model pollutants to investigate the photocatalytic activity of Cu–Cu_2_O@TiO_2_ nanocomposite under the visible light irradiation. Fig. 3c exhibits the UV-vis absorption spectrum of the MB aqueous solution with 5 mg of Cu–Cu_2_O@TiO_2_ as photocatalyst exposed to the visible light for different durations. And it is observed that the characteristic absorption peak of MB at 664 nm becomes weaker rapidly when the exposure time is extended. After about 180 minutes, the peak almost disappears. Meanwhile, the MB aqueous solution turned colourless gradually from beginning blue under the irradiation. This degradation efficiency of MB is much higher than that of the reported Cu_2_O@TiO_2_ composite or Cu–TiO_2_/RGO catalysts.^34,35^

Blank tests (without any catalysts or without light) were completed which exhibit a slight fall of the MB concentration (Fig. 3d). The results of the MB photodegradation with different photocatalysts under the same conditions are compared in Fig. S5.† After 180 minutes of the visible-light irradiation, about 12%, 11% and 30% of MB are decomposed catalysed by the pure commercial anatase nanoparticles, NOTT-100(Cu) and Cu_2_O obtained from the calcination of NOTT-100(Cu), respectively. These results also suggest that the Cu–Cu_2_O@TiO_2_ nanocomposite has a much more excellent photocatalytic activity, compared to either pure TiO_2_, Cu_2_O or NOTT-100(Cu) under the visible light irradiation (Table S1†). To further explore the practical applications of the Cu–Cu_2_O@TiO_2_ photocatalyst, the repeatability test was completed by recycling the sample three times in the degradation of MB under the same condition. In Fig. S6,† MB is found to be almost degraded entirely in each cycle, which suggests that there is no apparent decrease in photocatalytic activity in the three cycles. This result indicates that the Cu–Cu_2_O@TiO_2_ nanocomposite possesses good cycling stability for the photodegradation of MB.

When it was applied to the photodegradation of MO and 4-NP, Cu–Cu_2_O@TiO_2_ also shows good activity compared with pure TiO_2_ and NOTT-100(Cu). It is found that the characteristic absorptions of MO at 464 nm (Fig. S7†) decrease under the visible light irradiation. As shown in Fig. S8,† after irradiation for 3 h, residual MO and 4-NP drop to 38% and 62%, respectively.

**Scheme 2:**
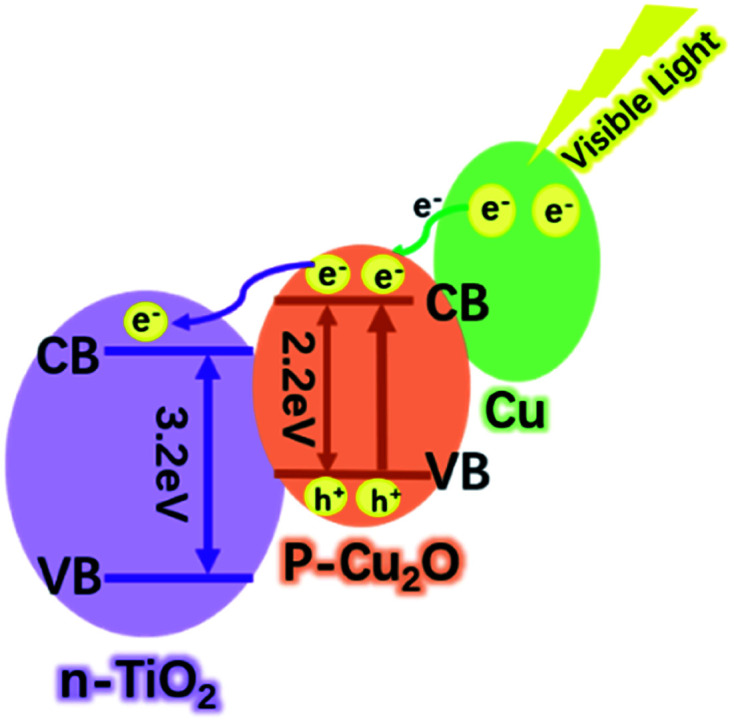
Photocatalytic mechanism of Cu–Cu_2_O@TiO_2_ nanocomposite under visible light irradiation.

There are many reasons for this excellent photocatalytic performance of the Cu–Cu_2_O@TiO_2_ nanocomposite, and the most crucial one can be ascribed to the efficient separation of the charge carriers at the closely combined Cu–Cu_2_O@TiO_2_ heterojunctions. Pure TiO_2_ nanoparticles can only be activated by ultraviolet light, whereas for Cu–Cu_2_O@TiO_2_ nanocomposite the photo-response range is widely extended. Therefore much more energy from the visible light can be utilised under the natural light irradiation. Moreover, the efficient separations of the photogenerated electron–hole pairs are driven by the heterojunction of p-type Cu_2_O and n-type TiO_2_ semiconductor. Although the TiO_2_ nanoparticles cannot be excited by the visible light, Cu_2_O can act as the primary absorber of visible light and be activated to generate electron–hole pairs. As shown in Scheme 2, the position of the conduction band (CB) in Cu_2_O is higher than that of TiO_2_, and so the active electrons (e^−^) in the CB of Cu_2_O tend to transfer to the CB of TiO_2_ when irradiated by the visible light. The photo-generated holes can effectively migrated to and accumulated at Cu_2_O, and meanwhile this will promote the acceleration of the photogenerated electron–hole separation in Cu_2_O and also curb the recombination of electron–hole pairs. In addition, the SPR electrons of Cu have enough energy to overcome the Schottky barrier formed on the Cu/Cu_2_O interfaces, thus the SPR effect of Cu excited by the visible light also enable the electrons transfer to the Cu_2_O.^36,37^ In the catalytic process, the excited electrons would migrate to the catalyst surface where they could be trapped by the absorbed oxygen, thereby generating ˙O^2−^ radicals. Then the oxidative H_2_O_2_ and ˙OH radicals can be produced by the reaction of those ˙O^2−^ radicals and H^+^. Finally, the organic pollutant molecules are oxidised and degraded by these strong oxidative radicals.^38^ Moreover, the porosity of the Cu–Cu_2_O@TiO_2_ nanocomposite should not be ignored as the high surface area can lead to better catalytic performance. Therefore, the Cu–Cu_2_O@TiO_2_ nanocomposite exhibits a remarkable photocatalytic performance under the visible light irradiation.

## 3. Experimental section

### 3.1 Materials and methods

XRD patterns were recorded on a Philip X'Pert with Cu-Kα radiation (*λ* = 0.154184 nm) at 40 kV and 50 mA with a scanning rate of 5° min^−1^. Scanning electron microscopy (SEM) was performed on FEI XL30 ESEM-FEG. Ultraviolet-visible (UV-vis) diffuse-reflectance spectra were analysed at room temperature by UV-2600 (Shimadzu, Japan) with BaSO_4_ and DI water as the references, which were converted to absorption spectra using the Kubelka–Munk method. Nuclear magnetic resonance (NMR) was recorded on Bruker Ascend TM 400; infrared spectroscopy (IR) was carried on ATR.

### 3.2 Synthetic procedures

#### 3.2.1 General procedure for synthesis of NOTT-100(Cu)

The solvothermal method was used for the synthesis of [Cu_2_(C_16_H_6_O_8_)(H_2_O)_2_]·2.5 DMF·4H_2_O. Firstly, a quantity of 400 mg of H_4_BPTC (1.21 mmol) and 832 mg of Cu(NO_3_)_2_·2.5H_2_O (3.58 mmol) were mixed and dispersed in DMF/ethanol/H_2_O (128 mL, 3 : 3 : 2 v/v/v) in a 250 mL round-bottom flask. Then CTAB (9.6 g, 26.34 mmol) was added to the mixture and mixed thoroughly. The suspension was heated with continuous stirring in a 65 °C oil bath for 24 hours, and a large amount of microcrystalline product precipitated. The blue crystalline product was washed with warm DMF (65 °C), H_2_O and acetone in turn, and dried briefly in the air. Yield: 745 mg (82.8%).

#### 3.2.2 Synthesis of NOTT-100@Ti(iv)

A quantity of 60 mg of NOTT-100(Cu) (807 μmol) was dispersed in 10 mL of *n*-hexane in a small glass vial with lid firstly. Then tetra-*n*-butyl orthotitanate (120 μL, 349 μmol) was dropwise added to the mixture and covered with lid rapidly. The suspension was continuously stirred for 24 hours. Finally, the blue product was separated by centrifugation at 8000 rpm for 2 min and dried briefly in the air.

#### 3.2.3 Synthesis of Cu–Cu_2_O@TiO_2_

A quantity of 60 mg of NOTT-100@Ti(vi) was placed in a small porcelain boat packing with tin foil paper, and then calcined at 550 °C for 4 hours (heating rate: 5 °C min^−1^) in the tube furnace. After calcination, the blue material turned into dark brown. Yield: 14 mg (23.3%). Elemental analysis: Cu_8_TiO_5.8_ (Cu, 78.47; Ti, 7.37%).

### 3.3 Photocatalytic experiments

The model organic pollutant, MB, was utilised to investigate the photocatalytic performance of samples under visible-light illumination. First, a quantity of 5 mg of the synthesised Cu–Cu_2_O@TiO_2_ was dispersed in 12.5 mL of deionised water (DI) (0.4 mg mL^−1^) *via* ultrasonication for 1 minute in a 60 mL glass beaker, and then 12.5 mL of a 4 × 10^−5^ M MB aqueous solution was added. A 300 W xenon lamp (CEL- HXF300, Beijing Aulight Co., Ltd., Beijing, PRC) served as the light source, equipped with a 400 nm cut-off glass sheet to filter out all the light at wavelengths below 400 nm. At the same time, the photocatalytic reactor and the visible light source was shielded by black cardboards during the reaction to remove the interference from outside light. Before illuminated by the visible light, the mixed solution of the photocatalyst and MB was under continuous magnetic stirring in darkness for 1 hour to establish an adsorption–desorption equilibrium. The suspension was also stirred during the whole irradiation process. At each time interval, 2 mL of the reaction liquid was taken out by a clean syringe, and the photocatalyst was removed by centrifugation at 10 000 rpm for 2 minutes. Then the clear MB solution was analysed by the Model UV-2600 spectrophotometer, with the measured maximum absorption at *λ*_max_ = 664 nm. NOTT-100(Cu), NOTT-100(Cu)@Ti(iv), TiO_2_ anatase nanoparticles, Cu_2_O after calcination of NOTT-100(Cu) were also applied into the degradation experiments of MB under the same experimental conditions for comparison purpose. For the degradation of MO and 4-NP, the experimental conditions are similar with MB, but with different initial concentrations. For MO, the concentrations of MO and the photocatalyst are 30 mg L^−1^ and 0.25 mg mL^−1^, respectively. For 4-NP, the fixed initial concentrations of 4-NP and the photocatalyst in the solution were 10 mg L^−1^ and 0.36 mg mL^−1^, respectively. All the photocatalytic degradation experiments were repeated three times.

## 4. Conclusions

In summary, the Cu–Cu_2_O@TiO_2_ mesoporous nanocomposite was successfully obtained by a facile two-step route, and its application in the photocatalytic degradation of organic pollutants under the visible light irradiation was utterly investigated. The Cu–Cu_2_O@TiO_2_ nanocomposite presents a lamellar Cu–Cu_2_O microsphere, embedded by numerous TiO_2_ nanoparticles. In the photodegradation of MB with the Cu–Cu_2_O@TiO_2_ nanocomposite, nearly 100% decolorization efficiency was achieved under the visible light in 3 h. The excellent photocatalytic property of the Cu–Cu_2_O@TiO_2_ nanocomposite can be ascribed to the unique mesoporous structure constructed from MOFs, the enlarged photo-adsorption range and also the efficient separation of the electron–hole pairs in its heterojunction. These characteristics indicate the Cu–Cu_2_O@TiO_2_ nanocomposite has huge potential for photodegradation of organic pollutants from water and the other environmental discharge.

## Conflicts of interest

There are no conflicts to declare.

## Supplementary Material

RA-010-D0RA01327G-s001
