# Supplementary material for: Mesoporous Cu–Cu2O@TiO2 heterojunction photocatalysts derived from metal–organic frameworks
Source: RSC Adv. 2020 Apr 9;10(25):14550–5. doi: 10.1039/d0ra01327g (PMC9052089; doi:10.1039/d0ra01327g)
Supplement: RA-010-D0RA01327G-s001 [file RA-010-D0RA01327G-s001.pdf]

Electronic Supplementary Material (ESI) for RSC Advances.
This journal is © The Royal Society of Chemistry 2020

Supplementary Information

Mesoporous Cu-Cu_2O@TiO_2 Heterojunction Photocatalysts Derived from Metal-Organic Frameworks

Wenling Zhao,[a] Chengcheng Liu,*[a]

[a] Institute of Molecular Sciences and Engineering, Institute of Frontier and Interdisciplinary Science, Shandong University, Qingdao 266237, P. R. China

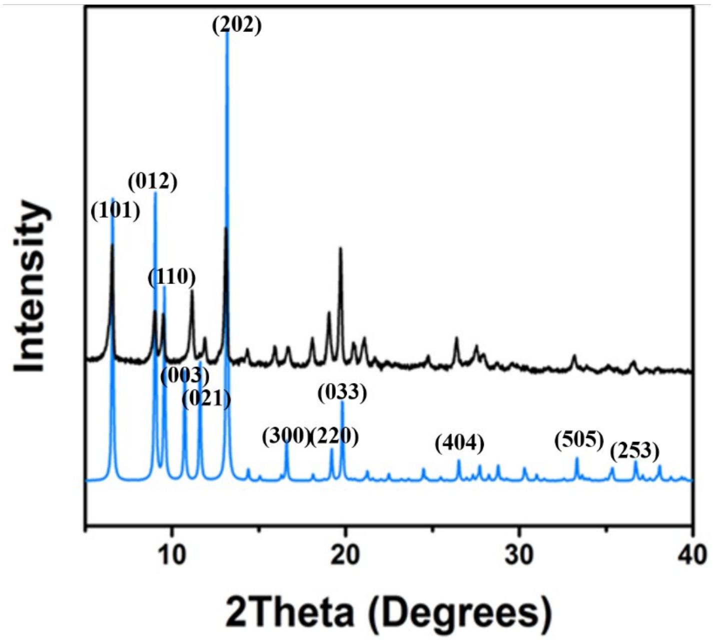

Fig. S1 XRD patterns of NOTT-100(Cu) (blue) and NOTT-100(Cu)@Ti(IV) (black).

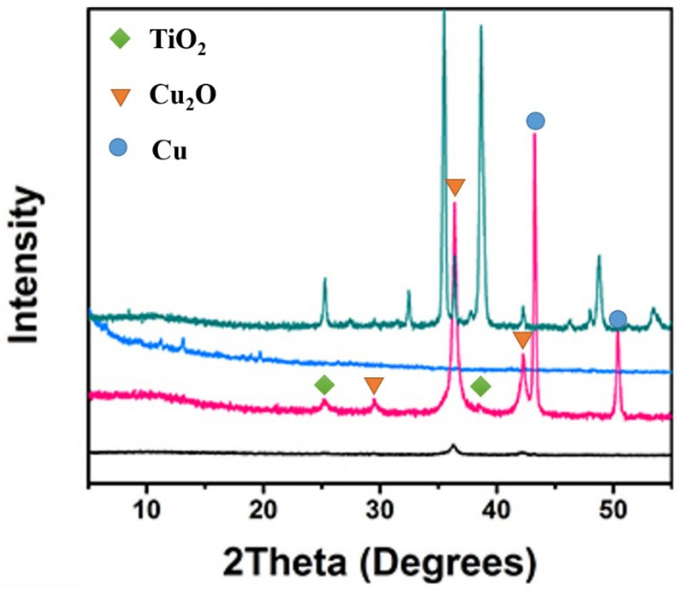

Fig. S2 XRD patterns of the samples with different calcination temperatures and time. 350°C for 2h (blue), 550°C for 2h (black), 550°C for 4h (pink) and 600°C for 4h (olive).

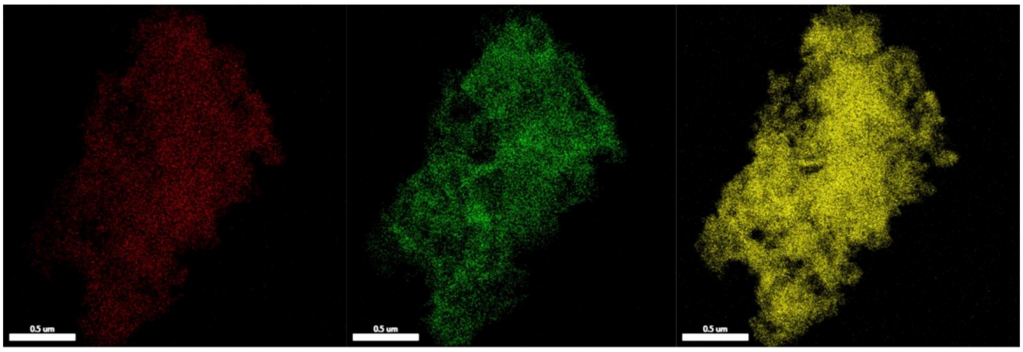

Fig. S3 EDS mapping of Cu-Cu_2O@TiO_2.

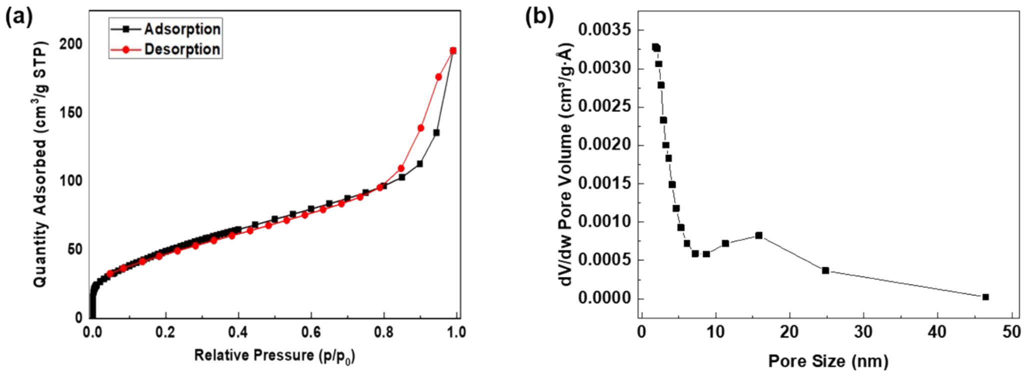

Fig. S4 N_2 adsorption-desorption isotherm (a) and BJH desorption pore distribution (b) of Cu-Cu_2O@TiO_2.

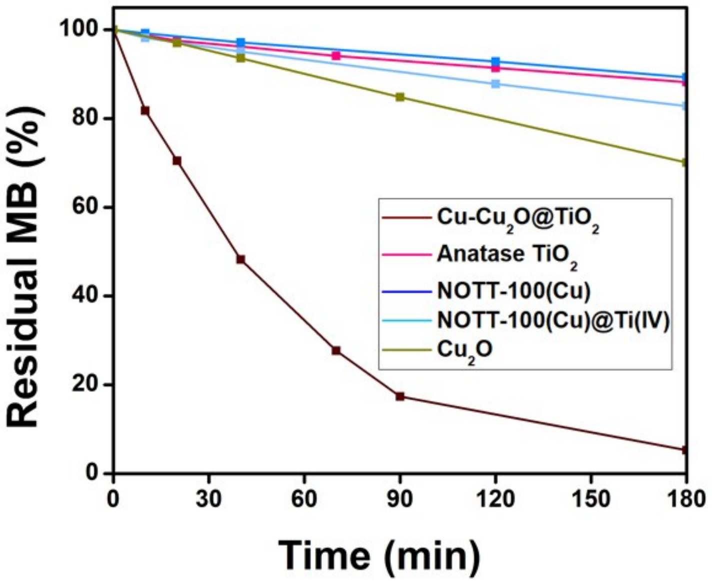

Fig. S5 Photodegradation of MB in the presence of Cu-Cu_2O@TiO_2 nanocomposite, commercial TiO_2 (anatase), NOTT-100(Cu), NOTT-100(Cu)@Ti(IV) and Cu_2O under the visible light irradiation.

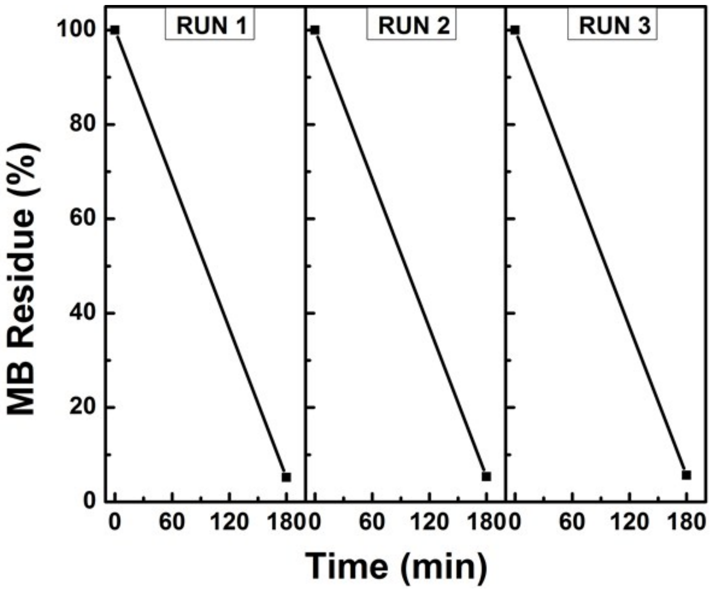

Fig. S6 Photodegradation of MB by Cu-Cu_2O@TiO_2 photocatalysts under visible light illumination for three runs.

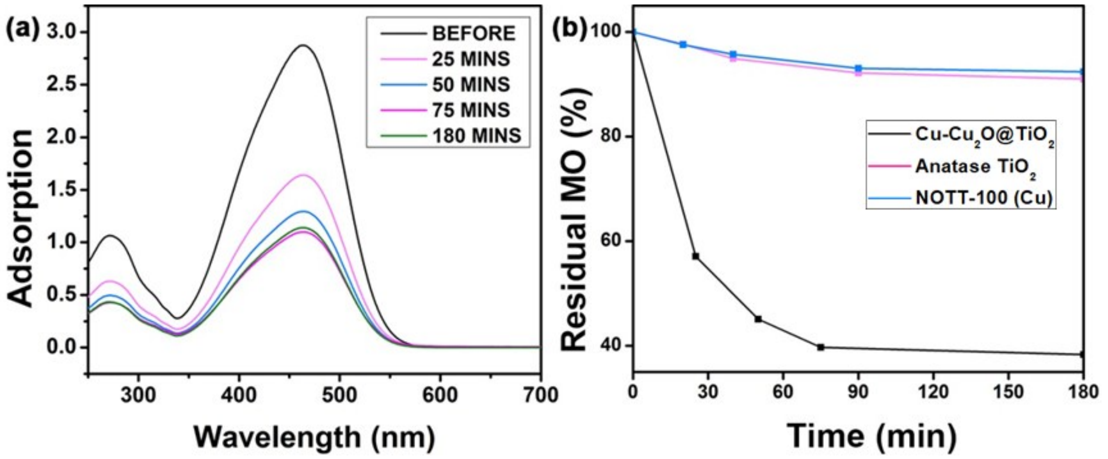

Fig. S7 (a) UV–Vis absorption spectra for MO solution in the presence of Cu-Cu_2O@TiO_2 nanocomposite; (b) Photodegradation of MO in the presence of Cu-Cu_2O@TiO_2 nanocomposite, TiO_2 (anatase) and NOTT-100(Cu) under the visible light irradiation

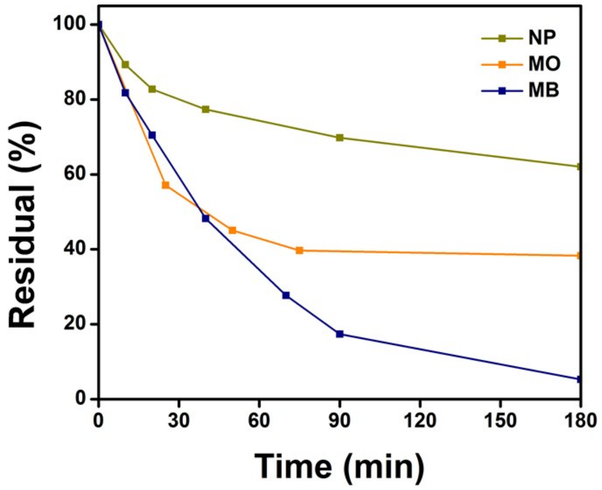

Fig. S8 Photodegradation of MB, MO and 4-NP using Cu-Cu_2O@TiO_2 nanocomposite under the visible light irradiation for 3 hours.

Table. S1 Decay rate values of dyes with Cu-Cu_2O@TiO_2, TiO_2 (anatase), and Cu_2O calcined from NOTT-100(Cu) under illumination with visible light

Dye	Catalyst	Decay rate (b) / min^{-1}
MB	Cu-Cu_2O@TiO_2	0.0166
MO	Cu-Cu_2O@TiO_2	0.0120
4-NP	Cu-Cu_2O@TiO_2	0.0062
MB	TiO_2 (anatase)	0.0007
MB	NOTT-100(Cu)	0.0006
MB	Cu_2O calcined from NOTT-100(Cu)	0.0023

The Langmuir–Hinshelwood kinetics model is used to determine the kinetics of the photocatalytic degradation rate, and the equation is as follows:

$$\ln\frac{C_0}{C} = bt + constant \qquad (1)$$

where C_0 is the original concentration of model pollutants before the light irradiation, and C is the concentration of model pollutants at different irradiation time. The apparent first-order rate constant b (min^{-1}) can be determined by the corresponding slope of $ln(C_0/C)$ and irradiation time t (min).
